# Outside‐In Medial Collateral Ligament Release Shows Superior Evidence for Safe Medial Joint Space Widening and Functional Recovery in Knee Arthroscopy: A Systematic Review

**DOI:** 10.1002/ars2.70087

**Published:** 2026-11-03

**Authors:** Hesan Rezaee, Mohammad Ayati Firoozabadi, Omid Salkhori, Mahdi Sahebi, Seyed Mohammad Javad Mortazavi

**Affiliations:** ^1^ Joint Reconstruction Research Center Tehran University of Medical Sciences Tehran Iran; ^2^ Department of Orthopedic Surgery Imam Khomeini Hospital Complex Tehran University of Medical Sciences Tehran Iran; ^3^ Students’ Scientific Research Center (SSRC) Tehran University of Medical Sciences Tehran Iran

## Abstract

**Purpose:**

To compare various medial collateral ligament (MCL) release techniques used during knee arthroscopy by evaluating their anatomical targets, biomechanical effects, medial joint space widening, clinical and functional outcomes, and associated complications.

**Methods:**

A comprehensive literature search was performed in PubMed, Scopus, and Web of Science for studies published between January 2000 and June 2025. Eligible studies focused on MCL release in adult knee arthroscopy patients and reported clinical and functional outcomes. Data on medial joint space opening, knee instability, functional scores, and complications were extracted.

**Results:**

Thirteen studies were included, with the outside‐in technique, especially the percutaneous pie‐crusting method, being the most extensively studied. The outside‐in technique consistently achieved the greatest joint space widening, with increases ranging from 2.5 to 12 mm. The inside‐out technique showed moderate joint space expansion (3‐7 mm). Functional outcomes improved significantly across all techniques. The outside‐in technique reported a Lysholm score increase from 48.2 to 80, a visual analog scale score decrease from 5.6 to 1.8, and an International Knee Documentation Committee score rise from 51.4 to 82.2. The inside‐out technique showed a moderate improvement in Lysholm score from 59.3 to 85.7, with no significant differences in instability or long‐term complications. Subperiosteal release also enhanced Lysholm scores from 50 to 92.5, with minor increases in joint laxity that resolved over time. All techniques showed low complication rates, with transient needle tract pain and mild ecchymosis being the most common side effects.

**Conclusions:**

MCL release techniques, especially the outside‐in percutaneous pie‐crusting method, improve medial joint space visualization and medial joint space during knee arthroscopy, aiding meniscal repair, partial meniscectomy, meniscal root repair, and other procedures. These techniques are generally safe but cause temporary ligamentous laxity similar to a grade I MCL sprain, so they are not entirely “benign.” Surgeons must weigh inducing controlled trauma against potential drawbacks of a tighter joint because the risk‐benefit balance varies case by case.

**Level of Evidence:**

Level IV, systematic review of Level III and IV studies.

## Graphical abstract

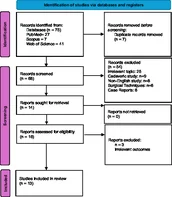

Knee arthroscopy is a critical procedure for diagnosing and treating meniscal injuries, particularly when the medial compartment of the knee is tight and restricted. In these cases, accessing the posterior horn of the medial meniscus can be extremely challenging.^1^ A restricted joint space may lead to difficulties in visualization, prolonged operative time,^2^ and increased risk of iatrogenic injuries,^3^ such as chondral damage or excessive meniscal resection. This can ultimately compromise clinical outcomes.^4^ The medial collateral ligament (MCL) is the primary stabilizer of the knee against valgus stress, with the superficial MCL (sMCL) playing a significant role in providing stability.^5^ The MCL consists of superficial and deep components, with the sMCL providing most valgus stability. The sMCL has its proximal attachment slightly proximal and posterior to the medial femoral epicondyle and its distal attachment on the proximal medial tibia approximately 5 to 7 cm distal to the joint line. The deep MCL (dMCL), also known as the medial capsular ligament, is shorter and attaches directly to the medial meniscus and joint capsule. Together with other key medial structures—including the posterior oblique ligament (POL), the posteromedial joint capsule, and the semimembranosus corner—the MCL forms part of the posteromedial complex (PMC). The PMC functions as an integrated unit that provides medial and posteromedial stability to the knee, particularly during flexion and extension. Understanding this anatomical relation is crucial because the MCL release techniques reviewed in this study primarily target the superficial and deep components of the MCL. Although these techniques may secondarily affect adjacent PMC structures such as the POL or posterior capsule, they do not constitute a comprehensive “MCL release” in the broadest anatomical sense. Therefore, this review focuses on MCL‐targeted release strategies and their clinical implications, while acknowledging the broader context of the PMC.^6^

Performing an MCL release during knee arthroscopy is an essential procedure, particularly for patients with medial compartment osteoarthritis or those requiring meniscal repair. The MCL release is typically achieved through 3 main techniques: outside‐in, inside‐out, and subperiosteal release. The primary goal of these techniques is to improve the joint space, reduce pressure within the medial compartment, and preserve knee stability. However, these methods are not without risks, including potential knee instability, neurovascular injury, and chondral damage.^7^, ^8^, ^9^ Given the knee's complex anatomy and the variety of available techniques, it is crucial for clinicians to have a thorough understanding of these approaches to minimize risks and achieve the best possible outcomes.

Although the primary purpose of MCL release is to improve visualization of the medial compartment and facilitate safer arthroscopic access, several studies have reported postoperative improvements in pain and functional scores. These improvements are likely indirect and may reflect enhanced meniscal preservation, reduced iatrogenic cartilage injury, and more efficient surgical execution rather than a direct therapeutic effect of the ligament release itself. The purpose of this study was to compare various MCL release techniques used during knee arthroscopy by evaluating their anatomical targets, biomechanical effects, medial joint space widening, clinical and functional outcomes, and associated complications. It was hypothesized that, although the surgical approaches differ, the various MCL release techniques would produce comparable increases in medial joint space and maintain low complication rates without causing clinically significant long‐term instability.

## METHODS

The current systematic review was conducted in accordance with the Preferred Reporting Items for Systematic Reviews and Meta‐Analyses statement standards.^10^ The study has been registered with International Prospective Register of Systematic Reviews under registration ID CRD420251118751. We thoroughly explored current evidence on MCL release techniques during knee arthroscopy. The main goal was to compare various methods based on their anatomical approach, biomechanical effects, clinical outcomes, and potential complications, particularly in cases where the medial compartment is tight and visualization of the posterior horn of the medial meniscus is limited.

### Literature Search

A comprehensive literature search was conducted in PubMed, Scopus, and Web of Science to identify relevant studies published between January 2000 and June 2025. The search employed a combination of Boolean operators (AND, OR) and relevant keywords to ensure thorough results. Search terms included phrases such as “posteromedial release,” “MCL release technique,” “medial collateral ligament release,” “pie‐crusting MCL,” and “knee arthroscopy.” Boolean operators were used to combine these terms logically—for example, using “AND” to include studies with both terms and “OR” to expand the search to studies with either term. The detailed search strategy for each database is provided in the Supporting Information. In addition to the database search, gray literature was sought through resources such as clinical trial registries (e.g., ClinicalTrials.gov) and open‐access conference proceedings to capture studies not published in peer‐reviewed journals. Although the term “PMC release” appears in some literature, the techniques in this review primarily target the superficial and/or deep components of the MCL. A true PMC release would require more extensive dissection, including the POL, the posteromedial capsule, and possibly the semimembranosus corner.

### Eligibility Criteria

The study has established specific criteria for inclusion and exclusion. The inclusion criteria are as follows: original studies that investigated MCL release during knee arthroscopy in adult participants; studies reporting clinical, anatomical, biomechanical, or functional outcomes; and studies addressing technique‐related complications, visualization improvements, or meniscal management. Meanwhile, the exclusion criteria are cadaveric studies, case reports, conference abstracts, and non‐English publications.

### Techniques for MCL Release in Knee Arthroscopy

Several surgical maneuvers have been described to increase the intra‐articular space of the medial compartment during knee arthroscopy, thereby improving visualization and instrument maneuverability. Noninvasive options include valgus stress and external tibial rotation, which can temporarily expand the joint space.^11^ In addition to using valgus stress and external rotation maneuvers, the effect of arthroscopic positioning aids should also be taken into account. Specifically, tools such as the wrap‐around positioner or “C” clamp can significantly influence widening of the medial compartment during knee arthroscopy. Positioning the device closer to the knee joint line is especially crucial because it facilitates a more efficient valgus fulcrum, thereby optimizing the application of valgus stress. This adjustment can result in more precise joint space widening, enhancing the exposure of the medial compartment, particularly in cases with a tight medial compartment and limited visualization. Moreover, the angle of knee flexion plays a vital role in the success of these maneuvers. A more acute knee flexion angle improves the leverage of the positioning aid, thereby increasing the overall effectiveness of joint space widening and ultimately enhancing intraoperative visualization. Once portals are established and compartmental inspection is completed, a palpation hook is placed vertically to assess posteromedial access. If the opening measures less than 5 mm, MCL release is generally recommended to facilitate adequate exposure. Although all techniques share the same objective, they differ in execution (inside‐out vs outside‐in), the structures targeted (sMCL, dMCL, or POL), and the instruments used (18‐gauge needle, banana blade).^8^ The techniques for MCL release in knee arthroscopy are summarized in Table 1 for comparative purposes.

**TABLE 1:** Comparative Summary of Techniques for MCL Release During Knee Arthroscopy

**Technique**	**Approach/Instruments**	**Structures Released**	**Indications**	**Key Features and Variations**	**Advantages**	**Limitations/Risks**
Outside‐in	Percutaneous puncture with a 16‐ to 18‐gauge needle	POL, posterior 1/3 sMCL; occasionally dMCL	Arthroscopic procedures requiring medial compartment access, including meniscal repair, partial meniscectomy, and meniscal root repair, especially in cases with tight medial compartments	– Single puncture (“popping” sound) – Pie‐crusting (multiple punctures) – Proximal vs distal release	Simple, minimally invasive; adaptable to release needs	Risk of neurovascular injury if landmarks are misidentified; variable effectiveness depending on the puncture site
Inside‐out	Needle or blade through the anteromedial portal under arthroscopic visualization	dMCL, meniscocapsular ligament (posterior 1/3 medial meniscus)	Medial meniscal repairs, particularly when dMCL release is required for enhanced visualization of the posterior horn of the medial meniscus	Multiple punctures were made, whereas valgus stress was applied	Direct visualization; precise targeting of intra‐articular structures	Higher technical demand; risk of iatrogenic cartilage/meniscal damage
Subperiosteal	Small incision, elevation of the sMCL from the tibia	sMCL (tibial attachment)	Situations requiring substantial medial compartment access, such as complex medial meniscus repairs, or when other less invasive methods fail to provide adequate exposure	Performed 1‐2 cm medial to the tibial tubercle	Provides wide release; applicable when greater exposure is required	More invasive; longer operative time; potential morbidity at the incision site

dMCL, deep medial collateral ligament; MCL, medial collateral ligament; POL, posterior oblique ligament; sMCL, superficial medial collateral ligament.

### Outside‐In Technique

The outside‐in method involves percutaneous punctures on the posteromedial aspect of the knee under arthroscopic guidance, typically using 16‐ or 18‐gauge needles. Transillumination helps avoid injury to the saphenous vein and nerve.^12^ The release typically targets the POL and/or the posterior third of the sMCL at the joint line,^4^, ^9^, ^13^, ^14^, ^15^, ^16^, ^17^, ^18^ although some authors have extended this approach to the dMCL.^3^, ^8^, ^12^, ^16^, ^19^ Several adaptations of the outside‐in approach exist.

• *Basic technique*: A single puncture to release the sMCL, with a “popping” sound indicating ligament relaxation, suitable for minimal releases with good outcomes and few complications.
• *Pie‐crusting*: Multiple small punctures along the medial side to gradually reduce tension.
• *Proximal versus distal release*: Punctures may be placed proximally at the medial femoral condyle or distally in a submeniscal position on the MCL.^3^
• The “magic point,” located 2.8 cm distal to the adductor tubercle, 1.8 cm below the medial epicondyle, and 1.2 cm above the joint line, ensures accurate targeting of the MCL while avoiding neurovascular injury.^13^

### Inside‐Out Technique

The inside‐out technique involves inserting a needle or blade through an anteromedial portal, directed toward the medial side of the knee. With valgus stress applied, punctures are made into the dMCL or meniscocapsular ligament, typically in the posterior third of the medial meniscus. Multiple perforations are performed under continuous arthroscopic visualization to prevent damage to intra‐articular structures.^1^, ^11^, ^20^

### Subperiosteal Release

In the subperiosteal technique, the sMCL is elevated off the tibia through a small (2‐3 cm) incision, typically placed 1 to 2 cm medial to the tibial tubercle. Sharp dissection is performed to the level of the sartorial fascia, which is carefully lifted to prevent injury to the underlying sMCL. The ligament is then elevated subperiosteally from its tibial attachment to achieve release.^2^, ^7^

### Outcome Measures

Based on the variables most consistently reported in clinical studies of MCL release during knee arthroscopy, the outcomes assessed in this review included medial joint space opening, postoperative valgus instability, visual analog scale (VAS), functional outcome measures (Lysholm, International Knee Documentation Committee [IKDC], and Knee Injury and Osteoarthritis Outcome Score [KOOS]), and procedure‐related complications.^2^, ^9^, ^12^, ^14^, ^17^, ^18^ Because MCL release is performed as an adjunct to facilitate arthroscopic treatment of intra‐articular pathology, the clinical scores reported in this review reflect overall postoperative outcomes rather than the isolated effect of the release itself.

### Study Selection

All records were reviewed and screened independently by 2 reviewers (H.R., M.S.) based on their titles and abstracts, and only publications that met predefined inclusion criteria were selected. Regarding disagreement at the title/abstract review stage, we screened the full text to prevent premature exclusion of studies. If a discrepancy arose during the full‐text screening, consultation with another author (S.M.J.M.) was used to make the final decision.

### Quality of Evidence

The methodological quality of the included studies was assessed using the Joanna Briggs Institute critical appraisal tools for case series and case‐control studies.^21^ Studies were rated on a 10‐point scale independently by 2 reviewers (H.S., M.S.), where scores of 1 to 4 indicate low quality, 5 to 7 indicate moderate quality, and scores of 8 to 10 indicate high quality and a low risk of bias. Each reviewer evaluated all studies separately. Any discrepancies in scoring or study appraisal were resolved through discussion and consensus; when agreement could not be reached, a third reviewer (S.M.J.M.) was consulted to make the final determination. Given the qualitative nature of the Joanna Briggs Institute checklist scoring system and the relatively small number of included studies, formal inter‐rater reliability statistics (e.g., kappa coefficients) were not calculated. However, agreement between reviewers was high after independent assessment and consensus discussion.

## RESULTS

### Study Characteristics

A comprehensive search of PubMed, Web of Science, and Scopus yielded 75 studies. After the removal of 7 duplicates, 68 articles were screened by title and abstract, of which 54 were excluded because of irrelevance. The remaining 16 studies underwent a full‐text assessment, resulting in the exclusion of 3 articles based on their reported outcomes. Ultimately, 13 studies satisfied the inclusion criteria. The study selection process is summarized in Figure 1, after the Preferred Reporting Items for Systematic Reviews and Meta‐Analyses guidelines. Of the 13 included studies, 10 were case series (Level III of evidence), and 3 were case‐control studies (Level IV of evidence). The median quality score for the included studies was 9 (range: 2‐10) according to Joanna Briggs Institute tools (Tables 2 and 3). These 13 studies involved 592 patients and evaluated various MCL release techniques. The outside‐in technique was the most frequently studied, with 9 studies and 407 patients. The inside‐out technique was analyzed in 2 studies including 57 patients. The subperiosteal release technique was analyzed in 2 studies including 128 patients.

**FIGURE 1:**
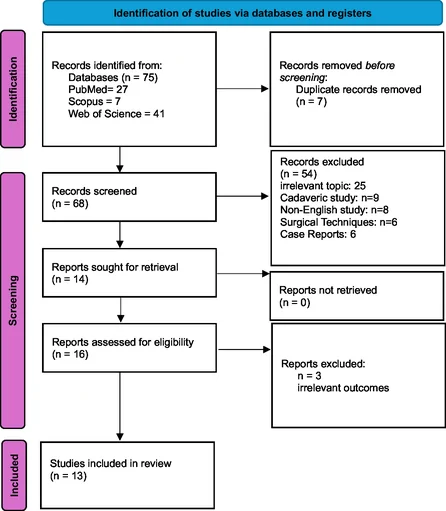
The flowchart of data screening for the present systematic review.

**TABLE 2:** JBI Case Series Questionnaire for Quality Evaluation

**Author**	**Were There Clear Criteria for Inclusion in the Case Series?**	**Was the Condition Measured in a Standard, Reliable Way for All Participants Included in the Case Series?**	**Were Valid Methods Used for the Identification of the Condition for All Participants Included in the Case Series?**	**Did the Case Series Have Consecutive Inclusion Session of Participants?**	**Did the Case Series Have Complete Inclusion of Participants?**	**Was There Clear Reporting of the Demographics of the Participants in the Study?**	**Was There Clear Reporting of Clinical Information of the Participants?**	**Were the Outcomes or Follow‐Up Results of Cases Clearly Reported?**	**Was There Clear Reporting of the Presenting Site(s)/Clinic(s) Demographic Information?**	**Was Statistical Analysis Appropriate?**	**Total Score (of 10)**	**Level of Evidence**
Fakioglu^9^	Yes	Unclear	Unclear	Yes	Yes	No	Yes	Yes	Unclear	Yes	6	IV
Javidan^11^	Yes	Unclear	Unclear	Unclear	Unclear	No	No	Yes	Unclear	No	2	IV
Chung^2^	Yes	Yes	Yes	Yes	Yes	Yes	Yes	Yes	Yes	Yes	10	IV
Lons^3^	Yes	Yes	Yes	Yes	Yes	No	Yes	Yes	Yes	Yes	9	IV
Polat^17^	Yes	Yes	Yes	Yes	Yes	Yes	Yes	Yes	Unclear	Yes	9	IV
Han^18^	Yes	Yes	Yes	Yes	Yes	Yes	Yes	Yes	Yes	Yes	10	IV
Arican^16^	Yes	Yes	Yes	Yes	Yes	Yes	Yes	Yes	Yes	Yes	10	IV
Akl^7^	Yes	Yes	Yes	Yes	Yes	Yes	Yes	Yes	Yes	Yes	10	IV
Butarbutar^20^	Yes	Yes	Yes	Yes	Yes	Yes	Yes	No	Yes	No	8	IV
Srisuwanporn^13^	Yes	Yes	Yes	Yes	Yes	Yes	Yes	Yes	Yes	Yes	10	IV

JBI, Joanna Briggs Institute.

**TABLE 3:** JBI Case Control Questionnaire for Quality Assessment

**Author**	**Were the Groups Comparable, Other Than the Presence of Disease in Cases or the Absence of Disease in Controls?**	**Were Cases and Controls Matched Appropriately?**	**Were the Same Criteria Used for the Identification of Cases and Controls?**	**Was Exposure Measured in a Standard, Valid, and Reliable Way?**	**Was Exposure Measured in the Same Way for Cases and Controls?**	**Were Confounding Factors Identified?**	**Were Strategies to Deal With Confounding Factors Stated?**	**Were Outcomes Assessed in a Standard, Valid, and Reliable Way for Cases and Controls?**	**Was the Exposure Period of Interest Long Enough to Be Meaningful?**	**Was an Appropriate Statistical Analysis Used?**	**Total Score (of 10)**	**Level of Evidence**
Claret^19^	Yes	Yes	Yes	Yes	Yes	Unclear	Unclear	Yes	Yes	Yes	8	III
Jeon^12^	Yes	Yes	Yes	Yes	Yes	Unclear	Yes	Yes	Yes	Yes	9	III
Gabr^14^	Yes	Yes	Yes	Yes	Yes	Unclear	Yes	Yes	Yes	Yes	9	III

JBI, Joanna Briggs Institute.

### Outcomes of Different Techniques

Across the reviewed studies, clinical and functional outcomes after different MCL release techniques were significantly improved. Most approaches resulted in measurable medial compartment widening, with joint space increases ranging from 2.5 to 12.5 mm. The techniques also improved arthroscopic visualization, with all approaches showing low complication rates, primarily involving transient needle tract pain and mild ecchymosis. However, no study to date has provided a comprehensive head‐to‐head comparison of all available techniques. A summary of the reported methods and their outcomes is presented in Table 4.

**TABLE 4:** Previous Study on Posteromedial Release in Knee Arthroscopy

**Author**	**Surgical Tech**	**Number of Patients**	**Clinical Outcome**	**Functional Outcome**	**Complication**
Fakioglu^9^	Outside‐in posterior third of the sMCL	18 patients	Radiographic joint space width: no differences between the preoperative and follow‐up (7.1 mm vs 7.2 mm)	Valgus stress with the knee in 30° of flexion: opening of <5 mm (compared with the contralateral) and a firm endpoint (grade I injury) lasting for 3.5 wk	All patients reported mild pain at the medial needle tract lasting for 15 d
Claret^19^	Outside‐in meniscectomy with or without pie‐crusting	140 (70 per group)	Meniscectomy + PC group: 1. Less pain at rest after 2 mo 2. Lower activity‐related pain at 6 mo	Meniscectomy + PC: higher scores on the Lysholm scale	No complication
Jeon^12^	Outside‐in release vs nonrelease	128 (64 per group)	No radiographic medial opening laxity	VAS score, Lysholm score, IKDC score: no differences	No complication
Lons^3^	Outside‐in	40 patients	Increase of 1° and 1 mm of medial opening under valgus stress instability	No functional instability	No complication
Polat^17^	Outside‐in	18 patients	Controlled release of the MCL in the knees provided 2.45 times wider visualization of the place	The final follow‐up Lysholm score increased significantly compared with the preoperative scores	2 patients (11%) showed grade I laxity on valgus stress with the knee in 30° flexion
Han^18^	Outside‐in	60 patients	Arthroscopic width of the medial space was larger than the prerelease (*P* < .01)	VAS, Lysholm, and IKDC scores showing significant improvement from preoperative scores	No residual valgus laxity of the knee
Arican^16^	Outside‐in pie‐crusting sMCL vs dMCL	sMCL: 34 patients	Intraoperative medial joint space opening: no significant difference between groups	KOOS score in both groups showed a statistically significant increase. No statistically significant difference was found between the groups	No complications
		dMCL: 33 patients			
Gabr^14^	Outside‐in proximal PC vs distal PC	34 proximal group	No radiographic medial opening laxity	VAS, KOOS, and IKDC were similar	No complications
		21 distal group			
Srisuwanporn^13^	Outside‐in	14 patients	At the 3‐mo follow‐up, no significant increase in the medial joint space width on valgus stress radiograph	‐	All patients reported slight pain at the medial needle tract of the affected knee for 2 wk
Chung^2^	Subperiosteal sMCL release	118 release group	In valgus stress tests (30°, 0°), at 3 mo, 12% and 2% of patients showed grade I laxity. Final follow‐up: 7% had grade I laxity in only 30° flexion	Lysholm and IKDC scores did not differ	Within 3 mo, 15% of patients experienced pain in the MCL attachment area
		20 nonrelease group		No residual valgus laxity	
Akl^7^	Subperiosteal sMCL release	30 patients	Stress valgus test: no difference was found between the normal and diseased sides	Lysholm score at follow‐up: significantly higher than preoperative	1. All of the patients complained of pain at the site of release after the operation, which lasted for 1‐2 wk postoperatively 2. Five individuals complained of postoperative ecchymosis
Javidan^11^	Inside‐out	35 patients	No case of chronic knee valgus laxity	‐	No complication
Butarbutar^20^	Inside‐out	22 patients	Intraoperative measurement of the medial joint space opening: 3‐7 mm (mean 4.04 ± 1.21)	‐	2 cases of mild needle scratches on the medial border of the femoral condyle

d, day; dMCL, deep medial collateral ligament; IKDC, International Knee Documentation Committee; KOOS, Knee Injury and Osteoarthritis Outcome Score; mo, month; sMCL, superficial medial collateral ligament; wk, week; VAS, visual analog scale.

### Intraoperative Medial Joint Space Opening

The degree of medial compartment opening is a key outcome measure of MCL release because it directly reflects the surgical objective of enhancing visualization and working space during arthroscopy. Clinical studies have quantified medial joint space widening using different techniques. The outside‐in technique has been the most extensively studied of all. Han et al.,^18^ in a retrospective series of 60 patients, reported that releasing the posterior third of the sMCL and the POL increased medial compartment height from 2.5 to 5.7 mm under arthroscopic assessment. Polat et al.,^17^ in a prospective study of 18 patients, revealed that the mean tibiofemoral widths were observed to be 5.0 mm in the neutral position, 7.5 mm with valgus stress at 30°, and 12.1 mm after pie‐crusting. Arican et al.^16^ compared the release of the sMCL (34 patients) and dMCL (33 patients), showing widening from 4.5 to 10.4 mm and from 4.3 to 11.2 mm, respectively, with no significant difference between groups (*P* = .83). Collectively, these studies confirm that outside‐in release consistently produces substantial intraoperative widening of the medial compartment. For the inside‐out technique, evidence is more limited. Butarbutar et al.^20^ reported on 22 patients undergoing dMCL release, with medial joint space widening ranging from 3 to 7 mm, corresponding to a mean intraoperative increase of 4.04 ± 1.21 mm. These findings confirm that the inside‐out approach also improves exposure, although the magnitude of opening appears smaller than in most outside‐in reports.

### Residual Knee Instability

Residual knee instability, defined as a subjective feeling of knee laxity or measured instability under valgus stress testing, was evaluated using a grading system based on the degree of joint opening. A grade I instability was defined as <5 mm of medial joint space widening with a firm endpoint, whereas grade II instability involved >5 mm of joint space widening with a soft endpoint.^22^ Concerns exist regarding the risk of medial instability after MCL release; however, most available evidence indicates that residual laxity or subjective instability is uncommon. The outside‐in technique is the most widely investigated, with 8 studies reporting outcomes. Fakioglu et al.^9^ prospectively followed 18 patients undergoing meniscectomy and observed that joint space widening (JSW) increased from 7.1 mm preoperatively to 9.1 mm at 1 week, 8.0 mm at 3 months, and 7.2 mm at 6 months (*P* < .001). By 6 months, JSW values were comparable to baseline, suggesting structural healing. Clinically, all patients showed <5 mm opening with a firm endpoint (grade I), which resolved within 3.5 weeks. Claret et al.^19^ compared 140 patients undergoing meniscectomy with or without pie‐crusting (70 per group) and found no valgus instability in the release group at 6 months, as assessed by both subjective and stress testing. Jeon et al.^12^ studied 128 patients (64 with release, 64 without release) who underwent meniscal repair and reported no significant change in valgus gap at 24 months in the release group (−0.1 ± 1.3 mm vs −0.1 ± 1.4 mm). Lons et al.^3^ evaluated 40 patients and noted a transient increase in medial opening at 6 weeks, with a 0.9 ± 1° increase in angle and a 1.1 ± 1 mm increase in height, both of which were statistically significant (*P* < .0001). Polat et al.^17^ followed 18 patients and observed grade I valgus laxity in only 2 cases at final follow‐up. Han et al.^18^ revealed that JSW increased from 5.9 ± 0.8  to 9.2 ± 1.1 mm at 1 week (*P* < .01) but normalized to 6.1 ± 0.9 mm at 3 months. Arican et al.^16^ compared the outside‐in release of the sMCL (34 patients) and dMCL (33 patients) and found no significant differences in valgus laxity angles at 12 months. Similarly, Srisuwanporn et al.^13^ reported no significant JSW changes at 3 months in 14 patients undergoing the magic point technique (7.42 ± 1.16 mm vs 7.47 ± 1.15 mm; *P* = .914). Collectively, outside‐in studies show that transient joint space widening is common early postoperatively, but it typically resolves within 3 to 6 months without leading to long‐term instability. The inside‐out technique has been less frequently studied but shows consistent outcomes. Butarbutar et al.^20^ followed 22 patients for 2 years and found no instability after release. Similarly, Javidan et al.^11^ evaluated 35 patients who underwent meniscectomy with pie‐crusting and reported no residual instability. The subperiosteal technique has been assessed in 2 studies. Chung et al.^2^ compared 118 release patients with 20 nonrelease controls and found that at 3 months, 12% showed grade I laxity at 30° and 2% at 0°. At final follow‐up, only 7% had grade I laxity at 30°, with no subjective instability. Akl et al.^7^ examined 30 patients and reported no significant differences in valgus stress testing between the operated and contralateral knees. Radiographs showed comparable medial openings: 2.27 mm versus 2.30 mm in extension (*P* = .317) and 3.27 mm on both sides in 30° flexion (*P* = 1.0). Taken together, outside‐in release has the strongest evidence base and shows only temporary increases in joint space without long‐term consequences. Inside‐out studies, although fewer, consistently show no instability. Subperiosteal release may be associated with low rates of mild, grade I laxity early on; however, no subjective or functional instability has been reported. Across all techniques, MCL release does not appear to result in clinically significant knee instability.

### VAS Score

Four studies reported pain outcomes using the VAS score, all employing the outside‐in technique. Overall, studies reported mixed pain outcomes after MCL release during knee arthroscopy. In most cases, performing an MCL release as an adjunctive procedure did not directly reduce pain, and some studies noted a transient increase in pain because of the needle punctures or the intentional low‐grade ligament sprain. However, the technique did facilitate the primary procedure (e.g., arthroscopic partial meniscectomy or meniscal repair) by improving joint space and intraoperative visualization, which may indirectly reduce iatrogenic pain from chondral injury. The transient increase in pain typically resolved within the early postoperative period, with significant pain reduction achieved later in the recovery phase, particularly in patients undergoing meniscal repair or meniscectomy. Claret et al.^19^ compared 140 patients undergoing meniscectomy with or without release (70 patients in each group). VAS scores during activity decreased significantly in both groups (*P* < .001), from 6 to 7 at baseline to 1 at final follow‐up. At 6 months, the release group reported significantly lower activity‐related pain compared with the nonrelease group (*P* = .003 and *P* = .031). At rest, VAS scores decreased from 2 at baseline to 0 to 1 at final follow‐up in both groups, with no significant between‐group differences. Jeon et al.^12^ retrospectively analyzed 128 patients (64 with release, 64 without release) who underwent meniscal repair. One day postoperatively, VAS scores were higher in the release group, although the difference was not significant. Both groups exhibited a steady decline in pain over time, and by 24 months, VAS scores were similar between the groups. Han et al.^18^ evaluated 60 patients undergoing MCL release (40 meniscoplasty, 20 meniscal suturing) and reported a significant reduction in VAS scores, from 5.57 ± 0.69 preoperatively to 1.80 ± 0.51 at final follow‐up (*P* < .001). Gabr et al.^14^ performed a retrospective matched study comparing 55 patients who underwent MCL pie‐crusting with concomitant anterior cruciate ligament reconstruction (ACLR) to 55 patients treated with isolated ACLR. Both groups showed significant improvement, with VAS scores increasing from 75 (50‐85) to 89 (80‐92) in the combined ACLR + Medial collateral ligament pie‐crusting group and from 70 (60‐84) to 85 (79‐93) in the isolated ACLR group. Subgroup analysis revealed a minimal benefit after proximal release (79 [63‐89] to 80 [71‐90]) and a greater improvement after distal release (70 [50‐81] to 82 [75‐90]), although no significant differences were observed between the groups.

### Lysholm Score

Seven studies reported Lysholm score outcomes after MCL release, including 5 that used the outside‐in technique and 2 that used the subperiosteal technique. Overall, all studies showed significant improvement from baseline, with long‐term comparisons showing no clinically meaningful differences between release and nonrelease groups. Among the outside‐in studies, Han et al.^18^ evaluated 60 patients and reported improvement from 48.2 ± 4.2 preoperatively to 80.1 ± 3.7 at final follow‐up (*P* < .001). Fakioglu et al.^9^ found that the median Lysholm score increased from 42 to 94 (*P* = .0002). Polat et al.^17^ observed a rise from 59.3 ± 7.9 (median, 60; range, 48‐73) to 85.7 ± 4.7 (median, 86; range, 78‐93) at final follow‐up (*P* < .001). Jeon et al.^12^ reported similar Lysholm scores at 24 months (85.1 ± 17.2 vs 83.9 ± 20.4, *P* = .7). Claret et al.^19^ studied 140 patients (70 in each group) and reported significant improvements in both groups. In the control group, scores rose from 54 ± 11 to 70 ± 4 at 2 months and to 92 ± 5 at 6 months, whereas in the release group, scores increased from 60 ± 9 to 73 ± 4 and to 92 ± 4 over the same period (all *P* < .001). Between‐group differences were significant at baseline (*P* = .001) and 2 months (*P* = .019), but not at 6 months (*P* = .095). Collectively, outside‐in studies show substantial functional recovery without evidence of long‐term disadvantage compared with nonrelease procedures. Two studies evaluated the subperiosteal technique. Chung et al.^2^ compared 118 release patients with 20 nonrelease patients and reported final Lysholm scores of 84.4 ± 12.1 versus 88.1 ± 12.8, with no significant difference (*P* = .117; 95% confidence interval, −2.18 to 9.47). Akl et al.^7^ observed marked improvement in 30 patients treated with subperiosteal release, with Lysholm scores increasing from 50.0 ± 8.1 (median, 49; range, 35‐65) preoperatively to 92.5 ± 3.9 (median, 93; range, 86‐98) postoperatively (*P* < .001).

### IKDC Score

Four studies reported IKDC outcomes after MCL release (3 using the outside‐in technique and 1 using the subperiosteal technique). Overall, all studies showed significant postoperative improvement, with long‐term results showing no clinically meaningful differences between the release and nonrelease groups. Han et al.^18^ reported a marked increase in IKDC scores from 51.42 ± 4.02 preoperatively to 82.17 ± 4.64 at final follow‐up (*P* = .00). Jeon et al.^12^ reported that IKDC subjective scores were similar preoperatively (42.0 ± 19.5 vs 46.6 ± 19.2) and remained comparable at 24 months (82.4 ± 19.3 vs 81.3 ± 20.1). IKDC objective grades also showed no significant between‐group differences (A: 67.2% vs 62.5%; B: 17.2% vs 28.1%; C: 10.9% vs 6.3%; D: 4.7% vs 3.1%). Similarly, Gabr et al.^14^ reported postoperative IKDC scores improving from 44 (32‐48) to 84 (70‐95) in isolated ACLR and from 58 (44‐67) to 90 (82‐97) in the combined group (Δ 40 vs 32), with no significant difference between groups. Within the Medial collateral ligament pie‐crusting subgroup, proximal and distal repairs showed comparable gains (47 → 81 vs 43 → 75; Δ 34 vs 32). Chung et al.^2^ reported final IKDC scores of 73.6 ± 11.2 versus 77.5 ± 11.9 (*P* = .112; 95% confidence interval, −1.43 to 9.24).

### KOOS Score

Two studies reported KOOS outcomes after MCL release using the outside‐in technique. Both showed significant postoperative improvement, with no significant differences between groups.

Gabr et al.^14^ reported substantial gains across all KOOS subscales, with no significant differences between groups (symptoms: 56 → 86 vs 71 → 91; pain: 58 → 94 vs 81 → 97; activities of daily living: 70 → 94 vs 91 → 96; sport/recreation: 25 → 81 vs 58 → 86; and quality of life: 25 → 75 vs 31 → 81). Within the Medial collateral ligament pie‐crusting group, proximal and distal release showed similar improvements (e.g., activities of daily living: 81 → 99 vs 56 → 83). Similarly, Arican et al.^16^ reported significant KOOS improvement from baseline to 1 year (sMCL: 65 ± 6 → 81 ± 6, *P* = .005; dMCL: 60 ± 8 → 82 ± 4, *P* = .002), with no significant difference between groups (*P* = .72).

### Complications

Across multiple studies evaluating MCL release, the procedure was generally safe with only minor, self‐limiting complications reported. Fakioglu et al.^9^ noted mild pain at the medial needle tract in all patients, which resolved within 15 days. Similarly, Srisuwanporn et al.^13^ observed slight medial needle tract pain lasting approximately 2 weeks, with no intraoperative or postoperative complications, including no iatrogenic chondral or neurovascular injuries. Akl et al.^7^ reported that all patients experienced pain at the release site for 1 to 2 weeks postoperatively and 5 developed ecchymosis, both of which resolved spontaneously. Butarbutar et al.^20^ documented 2 cases of mild needle scratches on the medial border of the femoral condyle without clinical consequences. Other studies also found no evidence of chondral damage or injury to the saphenous nerve or saphenous vein.

## DISCUSSION

This systematic review of 13 studies suggests that MCL release during knee arthroscopy may increase medial joint space, improve functional and pain outcomes, and be associated with mostly minor, transient complications without clear evidence of clinically significant long‐term instability. These findings indicate that MCL release can be a useful adjunct in patients with tight medial compartments because it appears to facilitate intraoperative visualization and instrument maneuverability while generally preserving postoperative knee stability. Among the available techniques, the outside‐in approach—particularly percutaneous pie‐crusting—has been the most frequently studied and shows the most consistent evidence of effectiveness, whereas the inside‐out and subperiosteal techniques also show favorable outcomes but are supported by more limited data. It is important to emphasize that although the term “PMC release” appears in some of the reviewed literature, the techniques evaluated in this systematic review primarily target the MCL—specifically its superficial and/or deep components. A true comprehensive PMC release would necessitate more extensive dissection involving the POL, the posteromedial capsule, and potentially the semimembranosus corner. The MCL‐focused releases reviewed here provide sufficient medial compartment widening for arthroscopic procedures, eliminating the need for more extensive PMC dissection and thereby minimizing surgical invasiveness while achieving the desired clinical outcomes. Among the various methods, the outside‐in technique, particularly the percutaneous pie‐crusting technique, has been the most rigorously studied. Across multiple high‐quality studies, this approach consistently resulted in significant medial joint space widening—often doubling the intraoperative joint width—along with transient increases in valgus laxity that typically resolved within 3 to 6 months postoperatively.^14^, ^17^, ^18^ Functional outcomes after outside‐in release were uniformly favorable, with substantial improvements observed in Lysholm, IKDC, and KOOS scores, often comparable to or superior to those seen in nonrelease cohorts. Several studies also reported reduced activity‐related pain and faster recovery of knee function in patients undergoing outside‐in release in conjunction with meniscal repair or meniscectomy.^3^, ^9^, ^12^, ^19^ Critically, no evidence of persistent instability was noted beyond the early postoperative period.

The inside‐out technique, although less commonly used, showed measurable improvements in medial compartment access and exhibited similarly low complication rates. The increases in joint space provided by this technique were sufficient for safe instrument maneuvering, with no adverse effects on postoperative valgus stability or functional outcomes. Although the magnitude of medial joint space widening was generally smaller compared with the outside‐in technique, the inside‐out method remains a viable alternative for cases requiring a more conservative release.^11^, ^20^

The subperiosteal release technique also yielded significant intraoperative widening and favorable long‐term functional outcomes. However, 2 studies noted the presence of transient grade I valgus laxity in a small proportion of patients early postoperatively. This mild instability resolved over time and did not contribute to subjective instability or functional impairment. Given its greater invasiveness compared with percutaneous techniques, subperiosteal release may be better suited for cases necessitating more extensive medial compartment access or where other methods fail to provide adequate exposure.^2^, ^7^

Complications associated with MCL release were generally rare, minor, and transient. These included medial needle tract pain, mild ecchymosis, and superficial scratches on the femoral condyle, all of which resolved spontaneously without long‐term effects. Importantly, no studies reported significant chondral damage or neurovascular injury, underscoring the safety of MCL release when performed by skilled surgeons with a comprehensive understanding of knee anatomy and careful technique. Although most studies observed transient increases in valgus laxity after MCL release, particularly with the outside‐in technique, the mechanism underlying the resolution of this transient laxity remains unclear and requires further investigation. One possible explanation is that the initial laxity is a temporary response to ligament relaxation and soft‐tissue healing. Because the knee stabilizes postoperatively, these changes may resolve within 3 to 6 months, potentially through tissue remodeling and scar formation. However, this hypothesis needs further research and supporting evidence. A clearer understanding of these processes could help refine surgical techniques and ultimately improve patient outcomes.

Based on the available evidence, the outside‐in MCL release, particularly the percutaneous pie‐crusting method, appears to be the most widely studied and may be considered a preferable technique for routine knee arthroscopy in patients with tight medial compartments. However, because of the lack of head‐to‐head comparative trials, this recommendation should be viewed with caution, and the choice of technique should be tailored to the individual patient's needs and the surgeon's experience. Future prospective, randomized, comparative studies with long‐term follow‐up are needed to establish definitive guidelines for technique selection and further refine the safety, efficacy, and optimal application of MCL release techniques in knee arthroscopy.

### Limitations

This systematic review has several important limitations. First, a quantitative meta‐analysis could not be performed because of the substantial clinical and methodological heterogeneity among the included studies, including differences in surgical technique, patient selection criteria, outcome measures, and follow‐up duration. Second, the overall level of evidence was limited because most included studies were case series or case‐control studies, with no high‐quality randomized controlled trials available for direct comparison of the different release techniques. Third, variations in reporting standards and incomplete data in some studies restricted the ability to perform subgroup analyses or draw definitive conclusions regarding the superiority of one technique over another. Fourth, no studies included in this systematic review reported the minimum clinically important difference, patient acceptable symptom state, or substantial clinical benefit for the MCL release techniques. The absence of these outcome measures limits the ability to assess the clinical significance of the observed improvements in joint space widening and functional outcomes across the various techniques. Fifth, the included studies differed in surgical indication, procedure type (e.g., meniscal repair, meniscectomy, or combined procedures), and patient characteristics; therefore, potential confounding factors could not be fully controlled, and the functional outcomes should be interpreted accordingly. Because this review was based on previously published studies, additional subgroup analyses—such as sex‐based comparisons—were not feasible because of the absence of disaggregated data and the relatively small sample sizes within individual reports. These factors should be considered when interpreting the findings of this review.

## CONCLUSIONS

MCL release techniques, especially the outside‐in percutaneous pie‐crusting method, improve medial joint space visualization and medial joint space during knee arthroscopy, aiding meniscal repair, partial meniscectomy, meniscal root repair, and other procedures. These techniques are generally safe but cause temporary ligamentous laxity similar to a grade I MCL sprain, so they are not entirely “benign.” Surgeons must weigh inducing controlled trauma against potential drawbacks of a tighter joint because the risk‐benefit balance varies case by case.

## SUPPORTING INFORMATION

Additional supporting information can be found online in the Supporting Information section.

## DISCLOSURES

The authors (H.R., M.A.F., O.S., M.S., S.M.J.M.) declare that they have no known competing financial interests or personal relationships that could have appeared to influence the work reported in this article.

## Supporting information

Supplementary Material
