# Supplementary material for: Outside‐In Medial Collateral Ligament Release Shows Superior Evidence for Safe Medial Joint Space Widening and Functional Recovery in Knee Arthroscopy: A Systematic Review
Source: Arthrosc Sports Med Rehabil. 2026 Nov 3:e70087. Online ahead of print. doi: 10.1002/ars2.70087 (PMC13729694; doi:10.1002/ars2.70087)
Supplement: Supplementary file 1 [file ARS2-9999-e70087-s001.pdf]

PM release

PubMed

(posteromedial release[Title/Abstract] OR posteromedial complex release[Title/Abstract] OR PMC release[Title/Abstract] OR medial collateral ligament release[Title/Abstract] OR pie-crusting[Title/Abstract]) AND (knee arthroscopy[MeSH Terms] OR knee arthroscopy[Title/Abstract])

WoS

TS= ((posteromedial release OR posteromedial complex release OR PMC release OR medial collateral ligament release OR pie-crusting) AND (knee arthroscopy))

Scopus

TITLE-ABS-KEY (posteromedial release OR posteromedial complex release OR PMC release OR medial collateral ligament release OR pie-crusting)
